# Shear bond strength between dental adhesive systems and an experimental niobium-based implant material

**DOI:** 10.1007/s10856-024-06834-7

**Published:** 2024-10-16

**Authors:** N. Brümmer, C. Klose, J-T. Schleich, H. J. Maier, M. Eisenburger, M. Stiesch, P.-C. Pott

**Affiliations:** 1 Department of Prosthetic Dentistry and Biomedical Materials Science Hannover Medical School Carl-Neuberg-Straße 1, 30625 Hannover, Germany; 2 Institute of Materials Science Leibniz University Hannover An der Universität 2, 30823 Garbsen, Germany

## Abstract

This study aimed to investigate adhesive shear bond strength (SBS) on an ultrafine-grained niobium alloy (UFG-Nb) that is a potential dental implant material. SBS of three adhesive systems combined with three composites to UFG-Nb was compared to corresponding SBS to Ti-6Al-4V and to zirconia. Specimens of the substrates UFG-Nb, Ti-6Al-4V and zirconia with plane surfaces were sandblasted with Al_2_O_3_, cleaned and dried. Three adhesive systems (Futurabond U, Futurabond M + , Futurabond M + DCA; all VOCO GmbH, Cuxhaven, Germany) were applied each on specimens of each substrate and light cured. One composite (BifixSE, BifixQM, GrandioSO; all VOCO GmbH) was applied and light cured resulting in 27 groups (*n* = 10) for all substrate-adhesive-composite-combinations. SBS was measured after 24 h of storage. To simulate aging equally prepared specimens underwent 5000 thermocycles before SBS measurement. There was no significant difference in SBS within the non-aged groups. Among the artificially aged groups, GrandioSO-groups showed a greater variance of SBS than the other composites. All significant differences of corresponding UFG-Nb-, Ti-6Al-4V- and zirconia-groups with same adhesive-composite-combination (ACC) were observed between UFG-Nb and zirconia or Ti-6Al-4V and zirconia but never between the two metallic substrates. The similarity between these materials might show in their adhesive bonding behavior. As there were no differences comparing corresponding groups prior to and after artificial aging, it can be concluded that aging does not affect SBS to UFG-Nb, Ti-6Al-4V and zirconia using the tested ACCs. Adhesive bonding of established ACCs to UFG-Nb is possible resulting in SBS comparable to those on Ti-6Al-4V and zirconia surfaces.

**Graphical Abstract:**

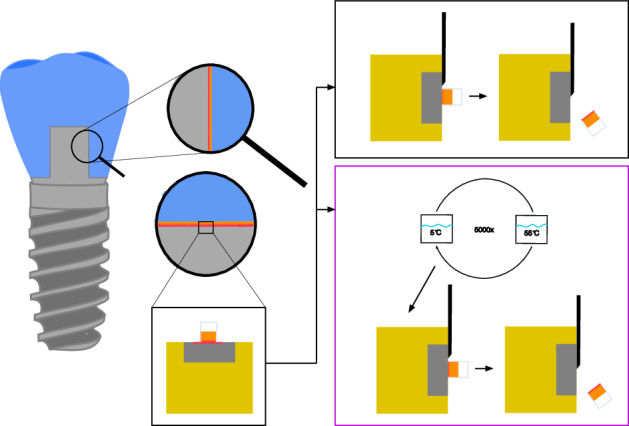

## Introduction

Dental implants are an integral part of prosthetic dentistry and the number of inserted implants increases constantly. Titanium and its alloys are the most common materials worldwide for dental implants [1]. The mechanical properties of titanium combined with its high biocompatibility resulting from a dense oxide layer on the surface make titanium a good choice for implants. Allergies to titanium are rare with a prevalence of 0.6%, on the other hand allergies in general have been expanding in society for unknown reasons [2]. In cases of material intolerances, zirconia is used as an alternative implant material to titanium. Although zirconia has good mechanical properties, implant fractures seem to appear more often than with titanium implants according to Sivaraman et al. [3]. Implant fractures are a severe complication because they usually lead to explantation. Their main risk factors are occlusal overloads, small implant diameters and bone loss due to periimplant infections [4]. Periimplantitis is an inflammatory disease of the periimplant tissue accompanied by a progressive loss of the implant supporting bone. With a prevalence of 22% [5], it is one of the most complex problems in modern dentistry. Besides the development of effective treatments for periimplantitis, new implant materials are needed to reduce implant failures due to mechanical instabilities and to provide suitable alternatives in case of allergies against titanium or its alloying elements, such as aluminum and vanadium in case of the widely used alloy Ti-6Al-4V.

Novel implant materials based on the refractory metal niobium seem to be promising in this context. Niobium is hypoallergenic, offers high biocompatibility caused by a dense oxide layer and has a good potential for osseointegration, which led to increased interest in implant material sciences [6–8]. However, the mechanical strength of pure niobium is not sufficient for its use as implant material [7]. Hence, the use of niobium is so far limited to implant surface coatings [6]. By the use of a materials processing technique called severe plastic deformation, the alloys’ strength can be increased without negative effect on the ductility of the material or the need for alloy constituents that might affect the biocompatibility. The result is a niobium alloy that has an ultrafine-grained microstructure, which leads to good long-term resilience against cycling mechanical loading and chemical influences. Moreover, the material retains the dense oxide layer that gives the material relatively high surface hardness and low conductivity, thus contributing to a good biocompatibility [8, 9].

Besides good mechanical and biological properties, due to different construction principles of fixed and removable dentures, dental materials nowadays must be able to achieve stable adhesive bonds to dental composites. Adhesive bonding allows the coupling of different materials in various clinical applications such as fixing crowns on teeth, attaching brackets for orthodontic treatments on teeth or ensuring a stable bond between metallic denture bases and resin denture parts [10–12]. Especially in implant dentistry it is necessary e.g. to fix ceramic crowns on patient individualized implant bases or abutments [13].

It has been well examined that the stability of the adhesive bond to titanium and zirconia results from a mechanochemical retention of the adhesive system on the roughened surface of the material [14–18]. Surface preparation by sandblasting with aluminum oxide is very effective to achieve optimal retention forces because it roughens the surface and improves the wettability by increasing the surface energy [14]. Further, aluminum oxide is partially remaining on the titanium surface and supports the chemical bond of adhesive systems, precisely of functional monomers, such as 10-Methacryloyloxydecryldihydrogenphosphat (10-MDP) [14, 15]. Its functional phosphoric acid group bonds directly to zirconia as well as to titanium oxide and other materials. The polymerizable group on the other hand allows the connection to composites [15, 19]. The shear bond strengths (SBS) of composite on sandblasted zirconia surfaces using adhesive systems that contain 10-MDP vary between 17.5 ± 1.3 MPa [16] and 57.6 ± 11.0 MPa [17]. In the literature in context of this study, a maximum SBS of 24.9 MPa on titanium surfaces is described [18]. Previous to this study, there were no data available on adhesive bonding on the new ultrafine-grained niobium alloy. Only a few studies proclaim a stable adhesive bond to other alloys with a considerable niobium fraction [20, 21]. The dense oxide layer on titanium surfaces has a high impact on adhesive bond stability. Considering that UFG-Nb also forms a dense oxide layer, the possibility for adhesive bonds to UFG-Nb surfaces is assumable as well.

The aim of this study was to investigate the bond strength between UFG-Nb and different adhesive-composite-combinations and to compare their bond strength to the substrates Ti-6Al-4V and zirconia. Therefore, the following three hypotheses were proposed:

1. The initial bond strength of UFG-Nb and dental adhesive-composite-combinations is independent from the adhesive-composite-combination.
2. The initial bond strength of the substrate-adhesive-composite-combination is independent from the substrate.
3. Artificial aging does not influence the bond strength of the substrate-adhesive-composite-combination.

## Material and methods

**Fig. 1:**
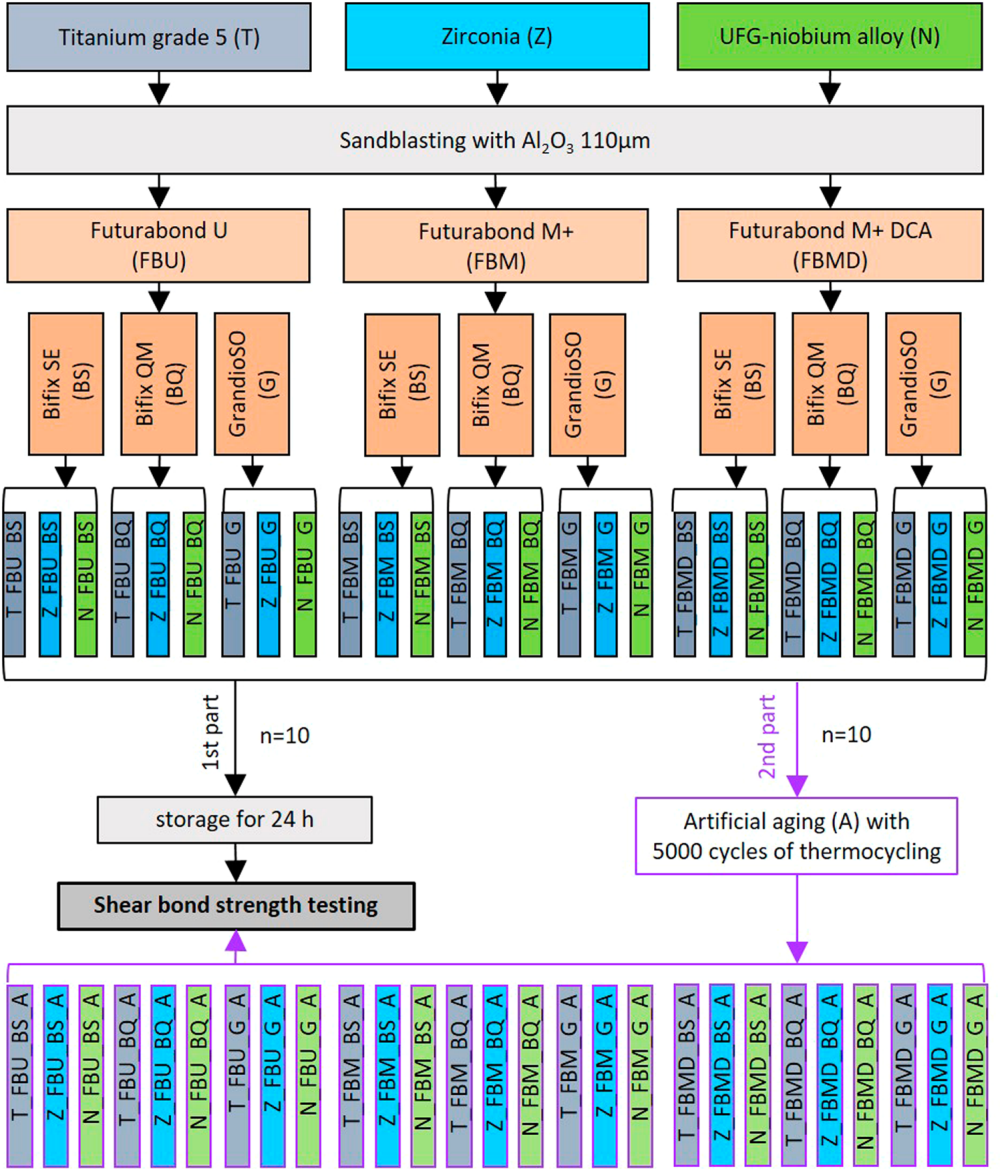
Flowchart of the experimental procedure

The study consists of two parts. The flowchart in Fig. 1 shows the detailed experimental procedure.

**Fig. 2:**
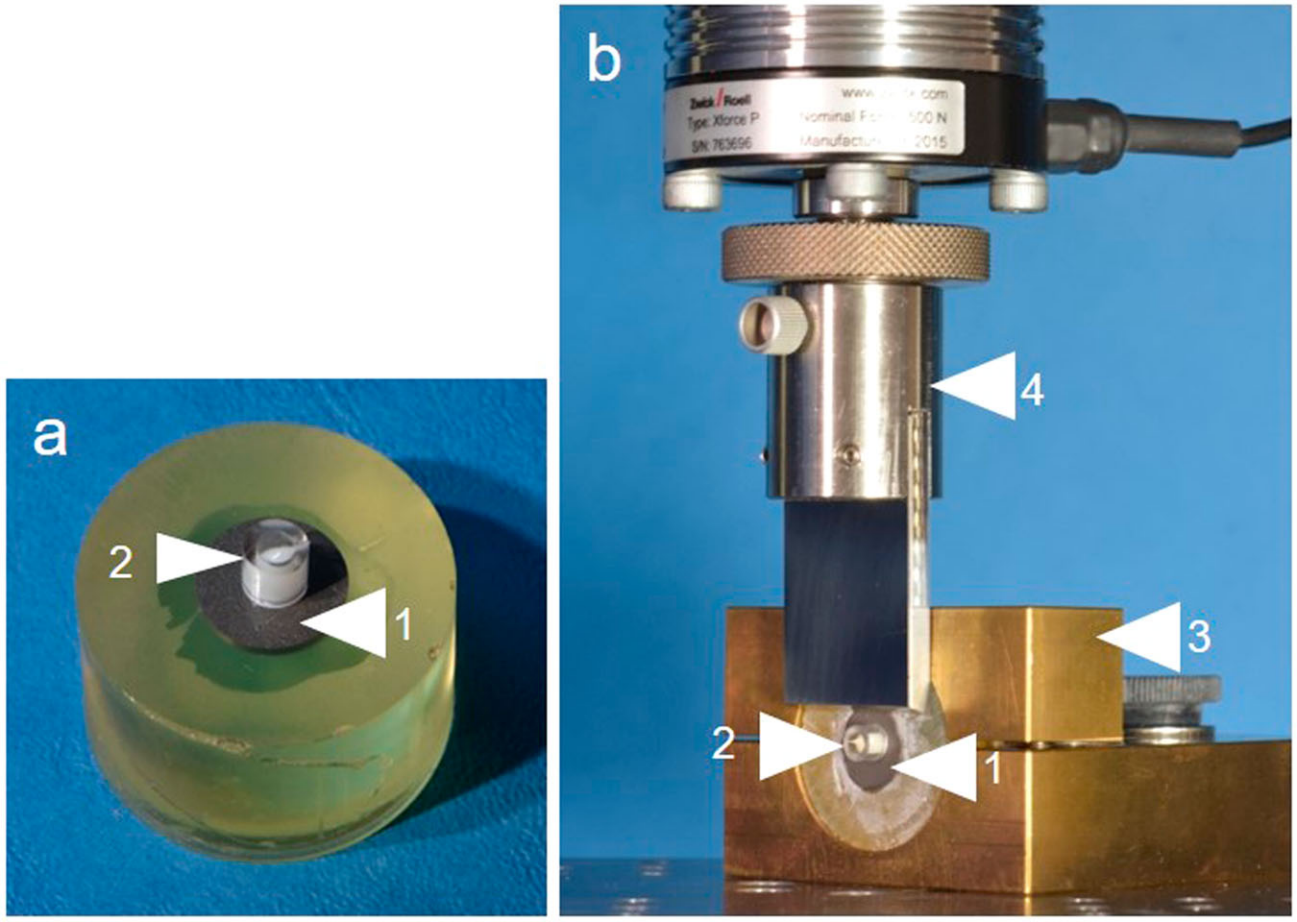
**a** Titanium specimen (1) with composite cylinder (2) (**b**) titanium specimen (1) with composite cylinder (2) fixed in specimen holder (3) for universal testing machine (4)

For specimen preparation, commercial Ti-6Al-4V discs with a diameter of 12 mm, commercial Y-TZP cylinders with a diameter of 12 mm and UFG-niobium alloy (UFG-Nb) cuboids with a surface of 4 × 4 mm and were embedded in epoxy resin. The UFG-Nb was produced and provided by the group of Maier et al. from Institute of Materials Science, Leibniz University Hannover, Germany [8, 22]. The niobium alloy examined in this study was an ultrafine-grained alloy of niobium and zirconia (Nb-1Zr). Remaining embedding compound was removed from the substrates surfaces by manual grinding on a diamond-coated disc. The material surfaces were sandblasted with 110 µm aluminum oxide at a pressure of 6 bar, cleaned with ethanol and dried with oil-free air. For the first part of the study, the three adhesive systems containing bifunctional monomers (Futurabond U, Futurabond M + , Futurabond M+ & Dual Core Activator; all VOCO GmbH, Cuxhaven, Germany) were applied with a single-use micro-brush each on 10 specimens of the three substrates according to the manufacturer’s instructions. The adhesive systems were light cured for 20 s using a polymerization lamp (Bluephase® G2, Ivoclar Vivadent, Ellwangen, Germany). Afterwards one of the three tested composites (Bifix SE, Bifix QM or GrandioSO; all VOCO GmbH) was applied using an acrylic glass mold to guarantee a defined bonding area of 3 mm diameter and was light cured for 60 s. To ensure complete curing of the dual cure composites the shear testing of the first parts groups took place 24 h after specimen preparation. Shear testing was performed in a universal testing machine (UTS 20 K, UTS Testsysteme GmbH & Co KG, Ulm, Germany) using a shear blade with a crosshead speed of 5 mm/min. To ensure a defined distance between the specimen’s surface and the shear blade the cylindrical specimens were fixed in a special specimen holder (Fig. 2).

For the second part of the study, specimens were prepared the same way as for the first part. 24 h after preparation all specimens underwent 5000 cycles of thermocycling between 5 and 55 °C with a dwell time of 30 s. Each cycle took 80 s. Shear testing took place 24 h after artificial aging. Due to limited availability of the niobium alloy the specimens had to be used multiple times, so that the initial condition had to be restored. Therefore, after shear bond testing remaining composite was removed by sandblasting the surfaces with 110 µm aluminum oxide before preparing the specimen for another testing group.

The statistical analysis of the data was done with IBM SPSS Statistics Version 26/27. Kolmogorov-Smirnov test confirmed the equality of variance and Levene’s test was used to prove the variances homogeneity. Differences between the groups were checked using a one-way ANOVA and following Post hoc Tukey Tests. The significance level was set at 0.05.

## Results

**Fig. 3:**
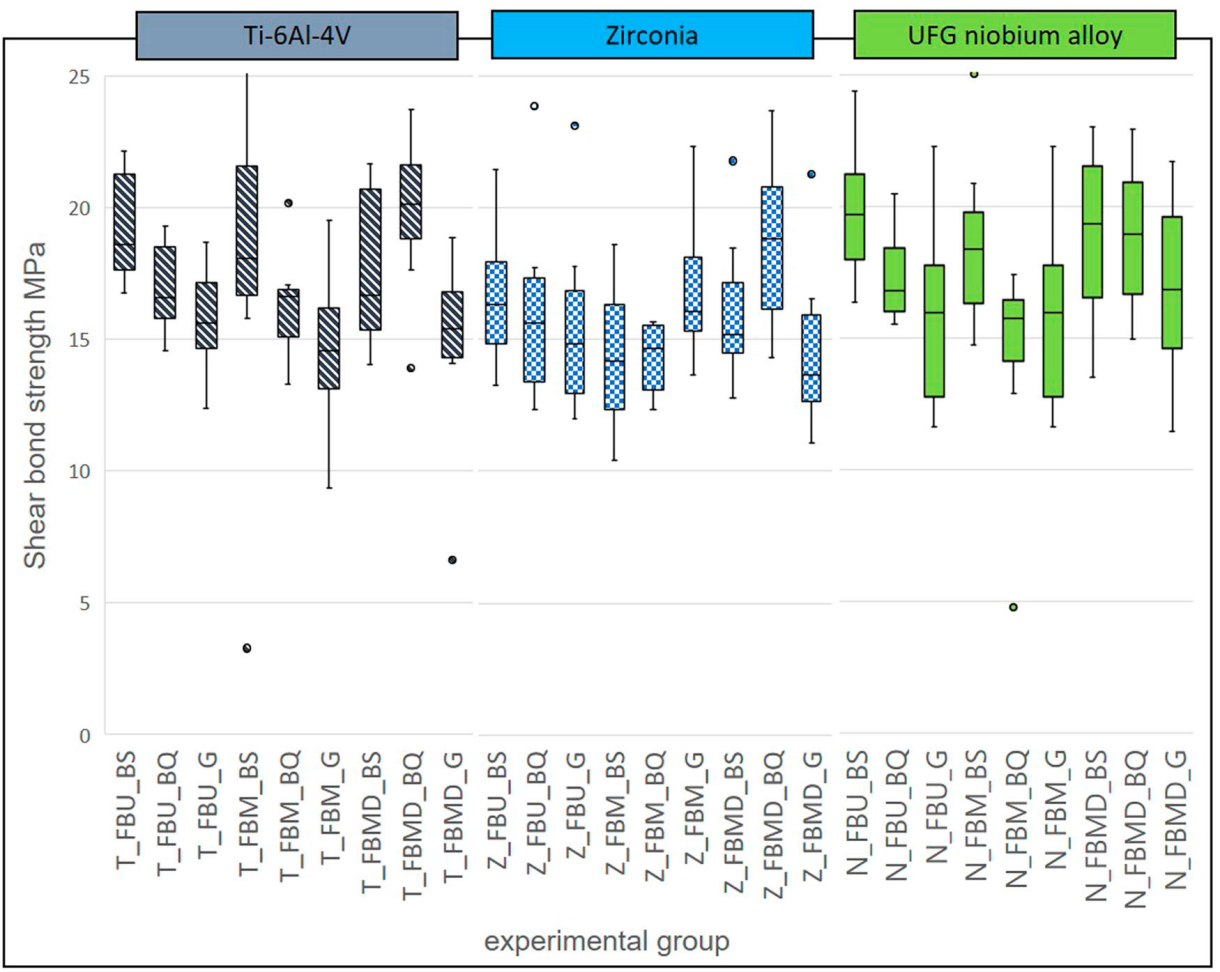
Boxplots of groups prior to artificial aging

The measured shear bond strength for the non-aged groups varied between 19.88 ± 2.72 MPa (T_FBMD_BQ) and 14.30 ± 1.26 MPa (Z_FBM_BQ). Between corresponding groups no significant differences were found (*p* ≥ 0,056). Figure 3 presents the results for all groups prior to artificial aging.

**Fig. 4:**
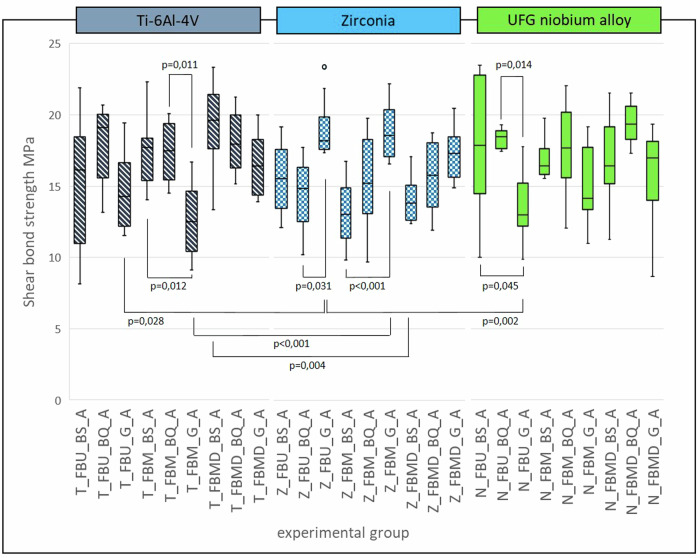
Boxplots of groups after artificial aging with *p* values of significantly differing groups

After artificial aging shear bond strength varied between 19.39 ± 1.37 MPa (N_FBMD_BQ_A) and 12.61 ± 2.41 MPa (T_FBM_G_A). Figure 4 shows the results for all groups after artificial aging. Within the titanium groups, the significantly lowest mean SBS was measured for group T_FBM_G_A (*p* ≤ 0.012). Within the zirconia groups group Z_FBU_G_A showed significantly higher SBS than Z_FBU_BQ_A (*p* = 0.031) and Z_FBM_G_A reached significantly higher SBS than Z_FBM_BS_A (*p* < 0.001). Comparing aged UFG-Nb groups, significantly lowest SBS was measured for group N_FBU_G_A (*p* ≤ 0.014). Comparing corresponding aged groups with the same adhesive-composite-combination but different substrates four significant differences were found. Group Z_FBU_G_A showed a significantly higher SBS than corresponding titanium group (T_FBU_G_A, *p* = 0.028) and corresponding UFG-Nb-group (N_FBU_G_A, *p* = 0.002). Z_FBM_G_A showed a significantly higher SBS than T_FBM_G_A (*p* < 0.001) and Z_FBMD_BS_A reached a significantly lower SBS than T_FBMD_BS_A (*p* = 0.004).

A comparison of SBS of the corresponding groups with the same adhesive-composite-combination showed no statistically significant differences before and after artificial aging.

## Discussion

The aim of the present study was to evaluate the bond strength of nine different dental adhesive-composite-combinations to an experimental niobium alloy in comparison to the most common implant materials titanium and zirconia [1, 23]. From literature, it is known that stable adhesive bond is achievable on both zirconia and titanium surfaces [12, 24–26]. In the present study, a maximum SBS of 19.88 ± 2.72 MPa (T_FBMD_BQ) was found on titanium. These results are in line with literature which reports SBS varying between 0.7 MPa [27] and 24.9 MPa [18]. Literature shows a large variability of SBS on zirconia surfaces between 17.4 ± 1.3 MPa [16] and 57.6 ± 11.0 MPa [17]. With a maximum of 18.67 ± 2.93 (Z_FBMD_BQ) on zirconia, comparable results were measured in this study. SBS of the UFG-Nb varied between 14.57 ± 3.66 MPa (N_FBM_BQ) and 19.75 ± 2.38 MPa (N_FBU_BS). There exist no comparable data for this material in literature. In our own previous studies on the adhesive bond to zirconia, bond strengths between 16.1 and 19.4 MPa [28] and between 8.6 and 19.1 MPa [29] were also found. These data are comparable to the results of the current study.

With focus on the SBS experiments prior to artificial aging, the individual comparison between groups of different adhesive-composite-combinations but same substrate did not identify any significant differences. Comparing the averages, most of the groups with GrandioSO tend to have lower SBS values than the other composites, except group Z_FBM_G (Fig. 3). However, this difference is not significant. Significant differences between corresponding groups of the same adhesive-composite-combination with different substrates also did not occur. Thus, the results of the experiments prior to artificial aging show that the bond strength values between the three substrates do not differ significantly and all nine tested adhesive-composite-combinations reach similar SBS values. Based on the results of the present study, the first hypothesis, stating that SBS to UFG-Nb is independent of the adhesive-composite-combination can be validated. The second hypothesis, stating that SBS of the substrate-adhesive-composite-combination is independent of the substrate material can also be validated based on the results of this study.

The current study is in line with other studies on adhesive bonding to materials and surface modifications. Adhesive bonds achieve a high stability when sandblasting the material’s surface with aluminum oxide and using adhesives systems that contain 10-MDP [13, 27, 30]. Apart from increasing the surface roughness and surface energy by sandblasting one beneficial effect of these methods is that 10-MDP can bond chemically to oxides that accumulate when sandblasting metallic surfaces with Al_3_O_2_ [31]. Having in mind that both titanium and niobium alloy develop a dense oxide layer this would also explain why the SBS values of the two metallic materials titanium and UFG-Nb are very similar.

In the second experimental part of the study samples were artificially aged by thermocycling. Thermocycling is a common method to simulate aging conditions in the oral cavity by applying hydrolytic and thermal stress on the materials. A negative influence of artificial aging on SBS values of adhesive bonds to zirconia is controversially discussed in literature [14, 28, 32]. In the present study, the comparison of SBS measured after artificial aging revealed significant differences within the substrate groups (Fig. 4). Especially combinations with GrandioSO showed high variability of SBS on the different substrates. Within the aged titanium groups as well as within the aged niobium alloy groups, significantly lower SBS were measured for groups containing the filling composite GrandioSO (T_FBM_G_A and N_FBU_G_A) compared to the corresponding groups with the other two composites. In contrast, the aged zirconia groups Z_FBU_G_A and Z_FBM_G_A showed higher SBS than one of the corresponding groups (Z_FBU_BQ_A / Z_FBM_BS_A). GrandioSO showed a tendency of lower SBS already before artificial aging. Literature supports the results, likewise claiming lower SBS of filling composites compared to resin cements when used on identically prepared zirconia surfaces [17, 29, 33]. GrandioSO has a higher viscosity compared to the other composites that were used here. For this reason, it might be more sensitive to application errors that can result in a smaller bonding area than the other, more flowable composites. This can explain the high variability in SBS. Comparison of corresponding groups after artificial aging with the same adhesive-composite-combination but different substrates revealed significant differences between either titanium and zirconia groups or UFG-Nb and zirconia groups. There were no significant differences between the groups of titanium and UFG-Nb. The similarity of these two metallic materials in contrast to zirconia might be the explanation for this observation.

To evaluate the influence of artificial aging on the bond strength corresponding groups with same substrate-adhesive-composite-combination were compared before and after artificial aging without finding significant differences between them. The negative influence of artificial aging on adhesive bond strength to titanium is discussed controversially. Cao et al. [25] and Yanagida et al. [30] found reduced SBS after artificial aging of adhesive bonds to titanium surfaces whereas Almilhatti et al. [15] and Tróia et al. [34] did not find any influence of artificial aging on the bond strength to titanium. With zirconia the majority of authors come to the conclusion that artificial aging has a negative influence on bond strength [14], but when surfaces are sandblasted and treated with an adhesive system containing 10-MDP the adhesive bond gets more resistant to artificial aging [28, 33]. The results of the present study underline this observation. Yanagida et al. could not find any long-term stable bond to titanium alloys and recommend the use of phosphate-based primers in combination with resins if necessary. However, they refer to the repair of denture base resins, which are exposed to different stresses than the materials examined here [35]. Nakamura et al. assumed that sandblasting titanium and zirconia with same size and pressure of Al_3_O_2_ results in less roughened zirconia surfaces in comparison to titanium due to the extreme hardness of zirconia. This could explain reduced SBS on zirconia in comparison to titanium [26]. In this study relative high pressure and a high grain size of 110 µm was used for surface preparation which might result in equivalent SBS values of zirconia compared to titanium and niobium alloy prior to and after aging simulation. The surface preparation might also explain the bond stability that was achieved on niobium alloy surfaces having in mind the similarities between titanium and the niobium alloy. Based on the results of the second part of the present study the third hypothesis, stating that artificial aging does not influence the bond strength of the substrate-adhesive-composite-combination can be validated.

## Conclusion

The aim was to investigate the adhesive bond strength of dental adhesive-composite-combinations to an ultra-fine grained niobium alloy (UFG-Nb), which is a promising implant material in comparison to zirconia and titanium. It can be concluded that stable bonds are achievable on UFG-Nb surfaces using the tested adhesive-composite-combinations. Moreover, the measured bond strength to UFG-Nb does not differ from zirconia and titanium surfaces. The adhesive bond to UFG-Nb is resistant to artificial aging. These findings mean a step on the way of establishing UFG-Nb as an implant material and must be complemented by further in vitro studies to simulate other clinical aspects.
